# Geodesic-Based Maximal Cliques Search for Non-Rigid Human Point Cloud Registration

**DOI:** 10.3390/s24216924

**Published:** 2024-10-29

**Authors:** Shuwei Gan, Guangsheng Xu, Sheng Zhuge, Guoyi Zhang, Xiaohu Zhang

**Affiliations:** School of Aeronautics and Astronautics, Sun Yat-Sen University, Guangzhou 510275, China; ganshw3@mail2.sysu.edu.cn (S.G.); xugsh6@mail2.sysu.edu.cn (G.X.);

**Keywords:** non-rigid registration, maximal clique search, human pose analysis

## Abstract

Non-rigid point cloud registration holds significant importance for human body pose analysis in the fields of sports, medicine, gaming, etc. In this paper, we propose a non-rigid point cloud registration algorithm based on geodesic distance measurement, which can improve the accuracy of the registration for matching point pairs during non-rigid deformations. Firstly, a graph is constructed for two sets of point clouds using geodesic distance measurement considering that geodesic distance changes minimally during non-rigid deformation of the human body, which can preserve the point cloud matching information between corresponding points. Furthermore, a maximal clique search is employed to find combinations of matching pairs between point clouds. Finally, by driving the human body model parameters, sparse matching pairs are overlapped as much as possible to achieve non-rigid point cloud registration of the human body. The accuracy of the proposed algorithm is verified with FAUST and CAPE datasets.

## 1. Introduction

The rapid advancement of 3D sensing technologies, such as range sensors, depth cameras, and multi-view reconstructions, has revolutionized the way human bodies are captured and represented in digital form. However, effectively utilizing these point clouds for applications such as biomechanics, virtual reality, and ergonomic assessments necessitates precise alignment and comparison of data captured from different instances or subjects. Non-rigid registration of point clouds is such a fundamental process in computer vision, utilized to align or match two or more sets of point cloud data [1]. Unlike rigid registration, which presumes that the shapes being aligned remain unchanged aside from translations and rotations, non-rigid registration must deal with complex deformations and variations in shape [2].

Since non-rigid deformation is point-wise, the transformation between two unstructured point clouds can be highly arbitrary. To address this complexity, one category of methods makes reasonable assumptions based on physical characteristics or domain knowledge to guide the estimation of deformation. These assumptions can enhance the accuracy and stability of registration while reducing the search space for potential solutions. Examples of such assumptions include the local rigidity assumption [3,4], the continuity assumption [5,6], the As-Rigid-As-Possible (ARAP) assumption [7,8,9], and the Coherent Point Drift assumption [6,10,11]. However, these assumptions have inherent limitations and are only applicable to specific scenarios, making these methods highly dependent on data quality and particularly suitable for handling large deformations.

Another category of methods addresses the 3D registration problem by generating correspondences free of outliers. These correspondences are crucial for defining non-rigid deformations, as they track how different parts of an object move and change shape over time. Typically, this approach involves two sequential stages: first, producing sparse correspondences, and second, performing optimization. The optimized energy function usually encompasses alignment components that measure the divergence between the two point clouds after transformation, alongside regularization components that ensure the transformation field remains smooth [12,13].

However, a significant limitation of these methods is their heavy reliance on the accuracy of the initial sparse correspondences. In the context of rigid point clouds, correspondence without outliers has been extensively researched due to the less stringent requirements of rigid transformations. Techniques involving maximum and maximal cliques with Euclidean distance can generate more accurate correspondences [14,15,16]. In these approaches, the preliminary set of correspondences is conceptualized as a compatibility graph, where each vertex represents a single correspondence and each edge signifies a pair of compatible correspondences. Subsequently, maximum or maximal cliques are identified within the graph, and a node-guided clique vetting procedure is employed to meticulously associate each graph vertex with the corresponding maximal clique that contains it.

In this paper, we introduce a three-dimensional human pose estimation method based on non-rigid point cloud registration. The fundamental assumption of our approach is that the topological structure among data points on the human body surface remains unchanged during motion and deformation, resulting in only minor variations in the geodesic distances between any two points. Geodesic distance represents the shortest path between two points on a curved surface or manifold. This metric is particularly crucial for capturing relationships within point clouds, especially during non-rigid deformations. Unlike Euclidean distance, which may overlook the surface complexity of deformable shapes, geodesic distance preserves the intrinsic geometry of the underlying manifold. This property makes it highly effective in accurately representing the relationships between points in scenarios involving non-rigid deformation, such as when modeling articulated objects or deformable surfaces. Our algorithm comprises two main components: a second-order spatial compatibility graph construction method based on geodesic distance measurements and a maximal clique search algorithm to obtain sparse correspondences.

**Figure 1:**
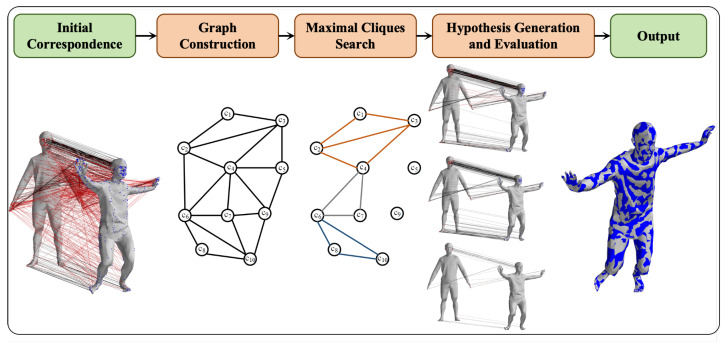
Workflow of geodesic-based maximal clique search for non-rigid point cloud registration.

For both the human body model and the point cloud data to be registered, we construct a second-order spatial compatibility graph where geodesic distances serve as weights. This graph effectively associates matching pairs that adhere to our core assumption among the initial correspondences. The maximal clique search algorithm is then employed to generate multiple sets of matching pairs that satisfy the constraint conditions. To refine these matching pairs, we utilize a nonlinear optimization algorithm derived from inverse dynamics to construct a loss function between the model and data matching pairs. This optimization process evaluates multiple outcomes by comparing the root mean square distance errors of neighboring points of the optimized results and the actual data. Ultimately, this procedure yields an effective set of matching pairs and accurate human body pose estimations. The workflow of the algorithm is illustrated in Figure 1.

The proposed geodesic-based maximal clique algorithm offers a distinctive advantage in non-rigid registration. By leveraging geodesic distances, which remain relatively stable even under large deformations, our algorithm improves the accuracy of sparse correspondences. The maximal clique search further enhances this by ensuring that the selected correspondences are highly reliable, addressing a critical limitation of current methods, especially during large point cloud deformation. In summary, the main contributions of our work are as follows:

- We propose a novel non-rigid point cloud registration method that leverages maximal clique searching within constructed graphs based on geodesic distance measurements. This innovative approach enables the accurate identification of correct correspondences in human point clouds by effectively filtering initial correspondences derived from simple feature matching. By utilizing maximal cliques, our method ensures robust correspondence selection, enhancing the precision and reliability of the registration process.
- We have designed an efficient, model-based optimization procedure that automatically filters the matching pair sets generated by maximal cliques. This optimization framework significantly enhances the robustness of our non-rigid registration method, particularly in scenarios involving large motions. By systematically refining the correspondence pairs, our approach minimizes errors and maintains high registration accuracy, even under substantial deformations and dynamic movements.

## 2. Related Works

Estimating human body poses from depth images or scanned point cloud data and performing point cloud registration have long been focal points of research in computer vision [17,18]. The data acquired from depth cameras and scanners fall under the category of unstructured 3D point cloud data. These unordered point clouds lack semantic information and cannot effectively describe the observed scenes or human body states. To extract meaningful information from these data, it is essential to align these point clouds with parameterized human body models, such as the SCAPE model [19], Frankenstein model [20], SMPL model [21], SMPL-X model [22], and others. Point-cloud-based human body pose estimation and reconstruction can typically be classified into two main categories: those based on geometric optimization [23] and those based on deep learning [24].

### 2.1. Geometric Optimization Algorithms

Geometric optimization algorithms iteratively reduce the matching error between two sets of point clouds by adjusting model parameters. These algorithms are characterized by their reliance on specifically designed objective functions and constraints based on various assumptions. They utilize optimization techniques to align models with point clouds and leverage different types of prior knowledge to reduce the computational complexity associated with high-dimensional point cloud data. Given two point clouds—one as the source and the other as the target—the goal of non-rigid registration is to find an appropriate transformation that deforms the source shape to align with the target shape [25]. Without prior model constraints, the transformation between two unstructured point clouds can be highly arbitrary. Therefore, reasonable assumptions based on physical characteristics or domain knowledge are often necessary to guide the transformation estimation, improving registration accuracy and stability while reducing the search space for potential solutions.

#### 2.1.1. Local Rigidity Assumption

The local rigidity assumption posits that, during deformation, point clouds remain relatively consistent within localized regions [3]. This assumption reduces the computational complexity caused by the large number of points in the cloud by constructing a deformation graph that binds local regions, significantly lowering computational overhead. For instance, the Non-Rigid Iterative Closest Point (N-ICP) algorithm [4] employs embedded deformation (ED) to describe deformations in different parts of the point cloud. Nodes in the deformation graph are adjusted through affine transformations to capture local point cloud position adjustments and deformations. This deformation model is integrated into the optimization process and combined with point-to-point correspondences between point clouds to gradually align them. However, N-ICP is sensitive to the initial estimate and often requires an initial alignment through rigid registration to achieve effective non-rigid registration, especially when dealing with significant global deformations.

#### 2.1.2. Continuity Assumption

The continuity assumption in non-rigid point cloud registration considers deformations between point clouds as continuous, facilitating the establishment of point correspondences and the estimation of deformation models for better alignment. The Coherent Point Drift (CPD) algorithm utilizes the principle of motion coherence in point cloud transformation, describing point correspondence using a Gaussian mixture model [5]. Through iterative optimization, CPD effectively handles point cloud deformations and non-rigid transformations. In 2021, Hirose reinterpreted the CPD algorithm within a Bayesian framework, ensuring convergence and eliminating the uncertainties present in the original algorithm [6]. This reinterpretation represents motion coherence as a prior distribution, providing a clearer and more explicit interpretation of its parameters.

#### 2.1.3. As-Rigid-As-Possible (ARAP) Assumption

The As-Rigid-As-Possible (ARAP) assumption [26] aims to maintain rigidity as much as possible during deformation, particularly for articulated deformations such as those of the human body [7]. The ARAP assumption minimizes excessive deformations and ensures that the relative positions between points on the same object do not change significantly. This assumption is particularly useful for point cloud registration of non-rigid shapes, especially when registering different poses of the human body, as the ARAP assumption ensures that the relative positions of the skeleton and key points remain consistent.

Geometric-optimization-based non-rigid registration methods offer flexibility and adaptability when dealing with various non-rigid deformations. They often do not require extensive prior knowledge or model construction, making them useful in specific scenarios. However, due to the lack of model constraints, these methods have high data quality requirements and are sensitive to noise. The choice of method depends on the specific application requirements and the quality of available data.

#### 2.1.4. Deep Learning Algorithms

Deep-learning-based 3D human body pose estimation using point clouds typically involves two main steps: extracting point cloud features with deep neural networks and regressing 3D joint coordinates or shape and pose parameters for human body models. Human body models, such as the SCAPE [19], Frankenstein [20], SMPL [21], and SMPL-X [22] models, are widely utilized as strong priors in point cloud registration and human pose estimation. These models, driven by predefined joint points, significantly reduce the degrees of freedom compared to non-model-based non-rigid point cloud deformations.

In Li et al.’s method [27] for estimating a 3D human body pose from a single RGB-D image, they first estimate 2D human body poses using a convolutional neural network (CNN) and derive 3D joint coordinates from the corresponding depth map. Subsequently, they optimize the SMPL model parameters and pose parameters by constructing loss functions based on both 2D and 3D joint point matching errors. However, the highly nonlinear relationship between the SMPL model parameters—which describe the local relative motion of human joints in an axis–angle format—and the point cloud data poses a significant challenge for directly regressing SMPL parameters from features extracted by backbone networks. To bridge this gap, several approaches have been proposed to establish an indirect relationship between point clouds and joint parameters.

For the VoteHMR algorithm introduced by Liu et al. [28], inspiration was drawn from the successful application of Hough voting in point cloud object detection. This method extracts sparse points from visible regions and establishes their relationships with skeletal joints. Cascaded joints are then used to obtain occlusion-aware joint features, which explicitly encode the human body pose and implicitly capture local geometric relationships. These features facilitate the estimation of shape parameters. Similarly, Wang et al. [29] proposed the Piecewise Transformation Fields (PTF) method, which learns to map any point from the pose space to a normalized pose space, thereby establishing local point alignment features. These features are subsequently used to estimate rotation parameters for a parametric SMPL model, offering greater accuracy and robustness compared to directly regressing SMPL parameters from global features.

Jiang et al. [30] introduced perceptual awareness of the human skeleton into the deep neural network framework by learning the mapping from point cloud features to skeleton features and then the mapping from skeleton features to SMPL parameters. Since point cloud features extracted by PointNet++ are unordered, the extraction process needs to follow the order of the skeleton. To achieve this, an attention module (AM) is incorporated to encode the spatial relationships of point cloud features. Additionally, a local graph aggregation (GA) module based on graph convolution is adopted to mine the local contextual relationships of point clouds without increasing memory requirements. Finally, a Skeleton Graph Module (SGM) based on graph convolution is employed to better learn features related to joint rotation parameters.

In Ying et al.’s work [31], RGB images are used to assist depth images in 3D human pose estimation. A 2D heatmap of human joints is first estimated and fused with a depth image to obtain a point cloud enriched with joint probability distribution information. This point cloud is then downsampled and input into a 3D network for learning and generating point-by-point features. A dense prediction module subsequently obtains the 3D coordinates of the human body’s joints.

Despite their effectiveness, these deep-learning-based methods for point cloud registration and human pose estimation require large numbers of training data, which limits their transferability to different datasets or real-world scenarios with varying data distributions.

## 3. Methods

### 3.1. Summary

Given a human model and a point cloud obtained from 3D scanning or multi-view RGB-D cameras, the goal of non-rigid point cloud registration is to align the two sets of point clouds—the source point cloud and the target point cloud—to structure the unorganized and unordered data effectively. Let $P^{s}$ and $P^{t}$ denote the two sets of points, respectively. The non-rigid point cloud registration method proposed in this paper employs a geodesic-based maximal clique search algorithm to establish sparse correspondences between articulated and deformed human body point clouds. This method leverages a manipulable human body model to construct an inverse kinematics loss function based on these sparse matching points. Through iterative optimization, the registration between the model and the data point cloud is achieved, resulting in an accurate estimation of the corresponding human body pose.

Specifically, the proposed method comprises two main components:

(1) Maximal Clique Search for Sparse Correspondence Generation

The first component involves identifying maximal cliques between the source and target point clouds, thereby generating multiple sets of matching pairs that adhere to geodesic distance constraints. This process can be further divided into three steps:

- Feature Extraction and Initial Matching: Extract features from both the source and target point clouds and perform feature matching to generate initial correspondences.
- Graph Construction with Second-Order Spatial Compatibility: Construct a compatibility graph using second-order spatial relationships among the matching pairs. The spatial compatibility measure is based on geodesic distances between point pairs.
- Maximal Clique Search: Search for maximal cliques within the constructed graph that satisfy the geodesic distance constraints, thereby generating robust sets of matching pairs.

(2) Inverse Kinematics Optimization for Pose Estimation

The second component quantitatively evaluates the generated maximal cliques by utilizing the matching pairs to constrain the inverse kinematics of the human body model. This involves obtaining the pose parameters of the human body model and assessing the alignment between the deformed model and the target data point cloud. The optimization process identifies the best-fitting correspondences, ensuring accurate pose estimation.

### 3.2. Geodesic-Based Maximal Cliques Search

#### 3.2.1. Correspondence Initialization

(1) Feature Extraction

First, for the given two sets of point clouds, it is necessary to detect key points or key features from the source and target point clouds. These key points are typically local maxima, curvature extrema, corners, edges, or other distinctive points within the point clouds. Commonly used point cloud features include Fast Harris 3D Point Features (FHPF), Signature of Histograms of Orientations (SHOT), and features extracted using deep learning.

(2) Feature Matching

Feature matching involves establishing correspondences between key points in the source and target point clouds, starting from the extracted features from the point clouds. Typically, nearest neighbor (NN) algorithms or other matching algorithms are used to identify points in the two point clouds with similar feature descriptors. Once matching key point pairs are found, correspondences are established between the source and target point clouds. Each point in the source point cloud is associated with a point in the target point cloud. In this paper, we use the FHPF to establish the initial features, and the distance between the features of each pair is evaluated to determine the similarity of the point pair.

#### 3.2.2. Second-Order Spatial Compatibility with Geodesic Distance Measurement

From Section 3.1, we obtain the initial point cloud correspondences $C={\left(c_{i}\right)}_{i=1,2,\ldots ,N}$, where $c_{i}=\left(p_{i}^{s},p_{i}^{t}\right)$ represents a set of correspondences between the source and target point clouds. In order to select the correct correspondences within the point cloud relationships, it is necessary to define an affinity between the various correspondences, resulting in the formation of a graph structure.

In graph structures, undirected graphs are typically represented as G=(V,E), where the vertex set is denoted as $V=\{v_{1},v_{2},\ldots v_{n}\}$, indicating the presence of n vertices in the graph. The edge set is defined as $E=\{\left(v_{i},v_{j}\right)|v_{i},v_{j}\in V)\}$ and consists of pairs of vertices. For any two vertices $v_{i}$ and $v_{j}$ in the graph, if there is an edge connecting them, then $(v_{i},v_{j})\in E$; otherwise, $(v_{i},v_{j})\notin E$. In an undirected graph, edges have no direction, which means that if $(v_{i},v_{j})\in E$, then $(v_{j},v_{i})\in E$, and both $\left(v_{i},v_{j}\right)$ and $\left(v_{j},v_{i}\right)$ refer to the same edge.

(1) Geodesic Distance Measurement

In point cloud registration, each correspondence $c_{i}$ represents a vertex in the undirected graph, and the relationships between vertices can be defined using different distance metrics. In rigid point cloud registration, common distance metrics include Euclidean distance, normal vector distance, and geodesic distance.

**Figure 2:**
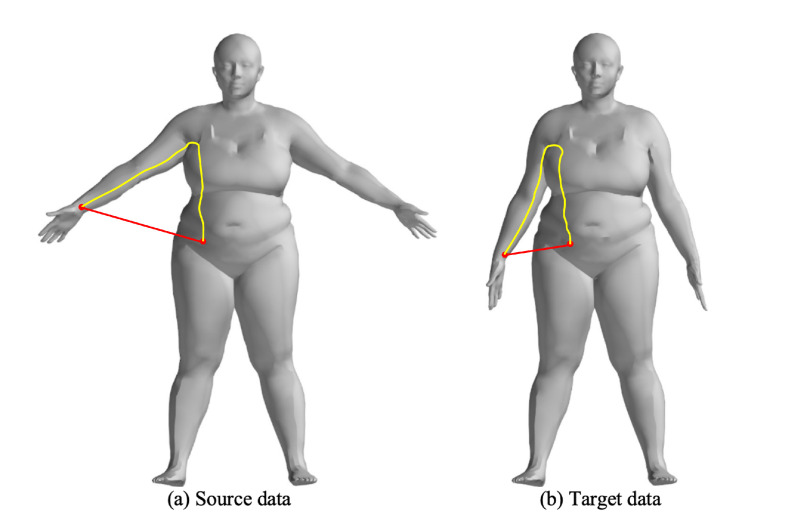
Illustration of the non-rigid deformation, the red and yellow lines represent the Euclidean and Geodesic distances between the wrist and waist points, respectively.

**Figure 3:**
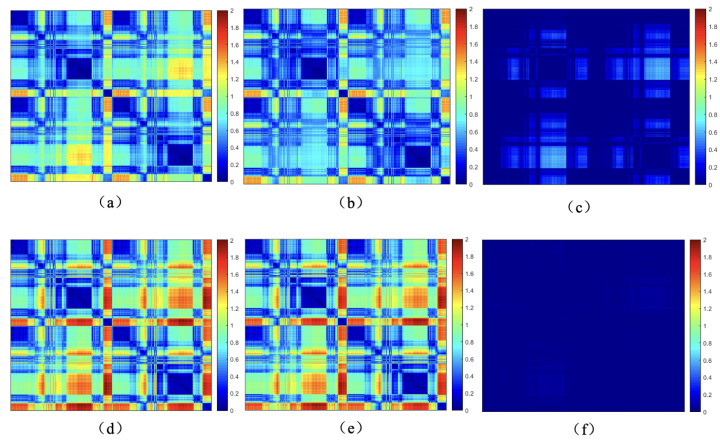
Euclidean distances, geodesic distances, and differences between matched points before and after human body motion, (**a**,**b**) are the Euclidean distance among source point and target point, respectively. (**c**) is the difference of (**a**,**b**). (**d**,**e**) are the Geodesic distance among source point and target point, respectively. (**f**) is the difference of (**d**,**e**).

For rigid transformations, Euclidean distance and normal vector distance are used as constraints in point cloud registration, similar to the metrics used in the Iterative Closest Point (ICP) algorithm. However, these metrics are not suitable for non-rigid point cloud registration, particularly for human body deformations, as they fail to capture the non-rigid deformation relationships. The complex movements of the human body lead to local relative deformations, as shown in Figure 2, which increase the number of parameters required for registration. In this case, the Euclidean distances between corresponding points (Figure 3a,b) also change due to non-rigid movements. Figure 3c shows the changes in Euclidean distances between corresponding points.

In this paper, we employ geodesic distances between point clouds to assess the spatial compatibility of two sets of correspondences, as shown in Equation (1). The fundamental idea is that, for any two correctly matched correspondences $c_{i}$ and $c_{j}$, the geodesic distance between the deformed original points $p_{i}^{s}$ and $p_{j}^{s}$ and the geodesic distance between the deformed points $p_{i}^{t}$ and $p_{j}^{t}$ should remain the same. Taking the non-rigid motion of the human body as an example in Figure 3, (d) represents the geodesic distances between points of the source point cloud, (e) represents the geodesic distances between points of the target point cloud, and (f) represents the difference in geodesic distances between corresponding points, which remains largely unaffected by non-rigid deformations.

(1) $$S_{geodist}\left(c_{i},c_{j}\right)=\left|D_{geodist}\left(p_{i}^{s}-p_{j}^{s}\right)-D_{geodist}\left(p_{i}^{t}-p_{j}^{t}\right)\right|$$

Computing geodesic distances on mesh surfaces is a fundamental problem in digital geometry processing and computer-aided design. Most existing exact algorithms partition the mesh edges into multiple interval windows, processing one window at a time. In this study, a vertex-oriented triangle propagation (VTP) algorithm is used to calculate geodesic distances, which is faster compared to conventional algorithms.

(2) First-order spatial compatibility

First-order spatial compatibility measurement suggests that the smaller the spatial distance difference between two matching pairs, the greater their similarity. Since the theoretically correct correspondence pairs (inliers) should have a distance difference of 0 between them, the similarity between two inliers is relatively high, forming a clustering effect among inliers. First-order spatial compatibility can be calculated using a distance metric. The operator is a monotonically decreasing function, meaning the smaller the distance metric between two matching pairs, the higher their spatial compatibility. Common monotonically decreasing functions include the negative exponential function:

(2) $$SC_{ij}=\exp\left(-\frac{S_{geodist}{\left(c_{i},c_{j}\right)}^{2}}{d^{2}}\right)$$

Here, *d* is the distance parameter.

(3) Second-order spatial compatibility

A second-order spatial compatibility measurement method was proposed in reference [32]. First-order compatibility suffers from ambiguity, meaning outliers can also have a high similarity with inliers. The first-order spatial compatibility measure is first binarized, and then, for any two compatible correspondence pairs, the number of correspondence pairs that are jointly compatible with them is calculated as their similarity. A hard thresholding is applied to the binarized first-order spatial compatibility, resulting in ${\left\{0,1\right\}}^{N\times N}$:

(3) $$C_{ij}=\left\{\begin{array}{cc} 0 & \mathrm{if}\;SC_{ij}\leq \tau_{cmp}\\ 1 & \mathrm{if}\;SC_{ij}>\tau_{cmp} \end{array}\right.$$

Here, $\tau_{cmp}$ is a hard thresholding parameter which can be set closer to 1 to reduce computational complexity for large point clouds. Since inliers are mutually compatible, the similarity between any two inliers is at least the number of inliers in all correspondence pairs, which is not true for inliers and outliers. Therefore, the second-order spatial compatibility measure can be calculated from the binarized first-order spatial compatibility matrix as follows:

(4) $$SC_{ij}^{2}=C_{ij}\cdot \sum_{k=1}^{N}C_{ik}\cdot C_{kj}$$

#### 3.2.3. Maximal Cliques Search

Once the spatial compatibility graph is constructed, the next step is to search for maximal cliques within the graph. A maximal clique is a subset of vertices C⊆V satisfying the following conditions:

- For every pair of vertices $v_{i},v_{j}\in C$, there is an edge $(v_{i},v_{j})\in E$, meaning that all correspondences in the clique are mutually compatible.
- The clique is maximal, meaning that no additional vertices from the graph can be added to the clique without violating the clique property (i.e., without breaking the spatial compatibility condition).

The maximal cliques represent sets of correspondences that are mutually compatible based on the spatial constraints, and thus are likely to be valid correspondences. The Eppstein algorithm uses a recursive backtracking framework similar to the Bron–Kerbosch algorithm but introduces optimizations that make it more efficient. The core idea is to explore candidate sets of vertices that may form maximal cliques and prune the search space to avoid unnecessary computations.

Define *R* as the current set of vertices that form a growing clique, *P* as the set of candidate vertices that can still be added to *R* without violating the clique property, and *X* as the set of vertices that have already been considered for the clique. At each step, the algorithm attempts to add a vertex from *P* to *R* and then recursively explores the potential cliques that can be formed. If a clique cannot be expanded any further, it is reported as a maximal clique.

The general recursion is structured as follows:

(5) MaxClique(R,P,X)

If both *P* and *X* are empty, then *R* is a maximal clique. For each vertex v∈P, recursively call the following:

(6) MaxClique(R∪{v},P∩N(v),X∩N(v))

where N(v) is the neighborhood of vertex *v* and the recursion continues until all vertices have been explored.

The finally obtained maximal cliques represent a consistency set, which is a collection of point pairs that are highly consistent under the graph constraints. To reduce the number of maximal cliques, only the maximal clique with the highest total weight is selected from all maximal cliques containing the same node, and duplicate maximal cliques are removed.

### 3.3. SMPL Inverse Kinematics with Sparse Correspondences

#### 3.3.1. Rigid Transform Estimation

In the process of human body deformation during motion, the range and variations of the limbs are more pronounced, while the motion of the torso is relatively small. Therefore, making an initial estimation of the rigid body motion of the entire human body based on the torso can effectively reduce the impact of the multiple degrees of freedom of the human body on non-rigid pose matching. The selection of torso points is based on the SMPL (Skinned Multi-Person Linear) model, which provides a well-defined human body mesh with a known number of vertices, each associated with specific body parts, including the torso, limbs, and head. In the SMPL model, the points (vertices) corresponding to the torso region are predefined, allowing us to extract these points for rigid body transformation estimation. To estimate the rigid motion, we select correspondences from the point cloud that match the torso vertices in the SMPL model. By leveraging the prior segmentation information from the SMPL model, we can reliably isolate the torso points for use in the rigid body transformation estimation, thus improving the robustness of the non-rigid registration process as follows:

(7) $$R_{0}=\arg\min_{R}\sum_{i=1}^{3}{∥{\hat{p}}_{i}-R{\hat{q}}_{i}∥}_{2}^{2}$$

In this equation, R represents the overall rotation of the human body, and $\left({\hat{q}}_{i},{\hat{p}}_{i}\right)$ denotes a pair of matching points in decentered coordinates. ${\hat{q}}_{i}=q_{i}-\frac{1}{N_{t}}\sum q_{i}$ and ${\hat{p}}_{i}=p_{i}-\frac{1}{N_{t}}\sum p_{i}$, where $N_{t}$ is the number of matching points in the torso. Expressed in matrix form, it can be written as follows:

(8) $$R_{0}=\arg\min_{R}{∥P-RQ∥}_{F}^{2}$$

The optimization problem for the Frobenius norm can be converted into an optimization problem for the trace of matrices as follows:

(9) $$\begin{array}{cc} \arg\min_{R}{∥P-RT∥}_{F}^{2} & =\arg\min\mathrm{trace}({\left(P-RT\right)}^{T}\left(P-RT\right))\\ \; & =\arg\max\mathrm{trace}(RTP^{T}) \end{array}$$

Denote $A=TP^{T}=U\Lambda V^{T}$, then $R_{0}=VU^{T}$.

#### 3.3.2. Non-Rigid Registration with Sparse Correspondences

In the process of non-rigid human point cloud registration, we utilize a Skinned Multi-Person Linear (SMPL)-model-driven optimization strategy to align the SMPL human body model with the point cloud data. The optimization is achieved by constructing a model–data matching loss function, designed to optimize the SMPL model’s pose parameters to match the observed point cloud, thus handling the non-rigid deformations effectively.

The overall loss function is defined as follows:

(10) $$E\left(\theta \right)=E_{v}+\lambda_{\theta_{b}}E_{\theta_{b}}$$

Here, $E_{\theta_{b}}$ represents the VPoser pose prior model, as introduced in SMPLify-X [22], which is used to regularize the human body pose and prevent unnatural deformations. VPoser learns a lower-dimensional representation of plausible human poses using a variational autoencoder (VAE), ensuring that the optimized pose parameters θ remain within the realm of realistic human motion. The term $\lambda_{\theta_{b}}$ is a weight coefficient that controls the influence of this regularization in the optimization.

The term $E_{v}$ is the matching loss between the model and the point cloud data. It measures the difference between corresponding points from the model and the data and is given by the following:

(11) $$E_{v}=\sum_{(p_{i},q_{i})\in C}\rho \left(M_{i}-p_{i}\right)$$

In this equation, $\left(p_{i},q_{i}\right)$ represents a sparse matching pair, where $q_{i}$ is a vertex on the SMPL model, and $p_{i}$ is the corresponding point in the point cloud data. After inverse kinematic (IK) iterations, $M_{i}$ represents the deformed position of the model point corresponding to $q_{i}$. The difference between the point cloud data point $p_{i}$ and the deformed model point $M_{i}$ is penalized using the Geman–McClure robust penalty function $\rho \left(s\right)=\frac{s^{2}}{s^{2}+\sigma }$, which is designed to mitigate the influence of outliers or noisy data by reducing the contribution of large errors.

To optimize the human body pose parameters θ, we use the L-BFGS algorithm, a quasi-Newton method well suited for high-dimensional optimization problems, including inverse kinematics. By iteratively minimizing the loss function, the algorithm aligns the SMPL model with the point cloud data, calculating nearest neighbor correspondences between the model points and the data points, and reducing the distances between matched pairs.

This SMPL-model-driven optimization strategy allows for accurate estimation of the deformed human body pose, even in scenarios with large deformations or rapid motion. By leveraging the predefined SMPL model, which includes anatomical knowledge of the human body, this method effectively reduces the impact of the body’s high degrees of freedom on the registration process, particularly in complex motion sequences.

After performing the maximal clique search and generating multiple sets of matching points between the model and the point cloud, the quality of each set of correspondences can be evaluated. One effective way to evaluate the matching accuracy is by using the nearest neighbor distance-based evaluation method, as shown in the following formula:

(12) $$D_{knn}=\sum_{i\in |M|}\min_{vinM(\theta )}{∥M_{i}-v∥}^{2}$$

In this formula, $D_{knn}$ represents the total sum of the squared distances between each point $M_{i}$ in the point cloud and its nearest neighbor v in the SMPL model M(θ), where θ represents the current pose parameters of the SMPL model. The term $\min v\in M(\theta )∥M_{i}{-v∥}^{2}$ computes the squared Euclidean distance between the point $M_{i}$ and its nearest point v in the deformed SMPL model.

This evaluation metric provides a quantitative measure of how well the points from the model and the point cloud align. By minimizing $D_{knn}$, we can ensure that the SMPL model is closely matched to the observed point cloud, indicating successful optimization.

## 4. Experiments

### 4.1. Datasets

(1) FAUST Dataset

The FAUST dataset (https://faust-leaderboard.is.tuebingen.mpg.de/download, accessed on 1 September 2023) consists of 300 high-resolution scans of real human bodies, involving 10 individuals and a wide range of poses and evaluation methods [33]. This dataset was collaboratively created by the Max Planck Institute for Intelligent Systems in Germany and the University of Padua in Italy. Its purpose is to provide a standard benchmark for evaluating and comparing various 3D human body reconstruction methods and mesh registration methods. For our experiments, we used the registration part of this dataset, which includes 100 different poses from various individuals in the form of triangular mesh data, to perform non-rigid registration and human body pose estimation experiments.

(2) CAPE Dataset

The CAPE dataset (https://cape.is.tue.mpg.de/download.php, accessed on 1 September 2023) is a large-scale human body dataset created in collaboration between the Max Planck Institute for Intelligent Systems in Germany, the University of Tübingen in Germany, the Swiss Federal Institute of Technology in Zurich, and the University of Grenoble Alpes in France. This dataset consists of eight males and three females and covers a wide range of human body motions. Approximately 80,000 frames of 3D scan sequences were captured at 60 FPS using a high-precision scanner from 3dMD. These raw scan data were then fitted with SMPL human body models, and clothed human body mesh data were obtained using nonlinear optimization algorithms [34].

### 4.2. Sparse Correspondence Evaluation

To validate the accuracy of the sparse matching pairs extracted by the GBMAC, this section includes an analysis of the experimental results obtained for both the FAUST dataset and the CAPE dataset. A comparison is made between the Global Matching Diffusion Pruning (DP) algorithm [35] and the Geodesic-Based Maximal Clique Non-Rigid Registration method (GBMAC) proposed in this paper for sparse matching on dynamic human body data.

In the analysis of the experimental results, the performance of DP and the GBMAC in generating sparse matching pairs for dynamic human body data from both the FAUST and CAPE datasets is compared and assessed.

#### 4.2.1. FAUST Dataset Sparse Correspondence Evaluation

**Figure 4:**
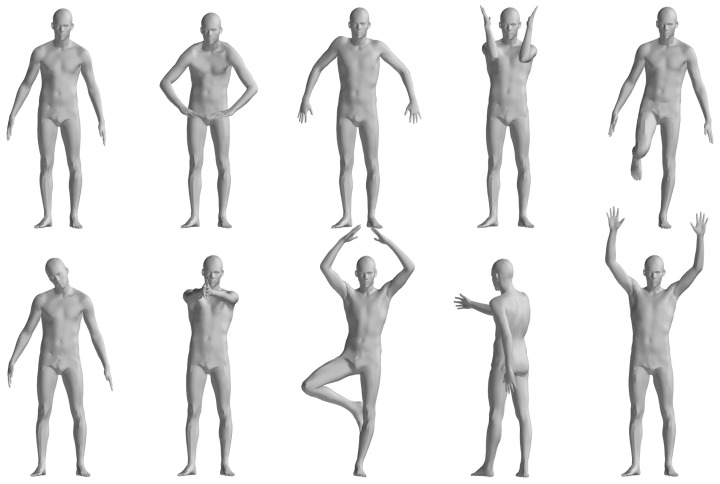
Illustration of FAUST dataset 01 Personnel Action [33].

The FAUST dataset’s registration section includes ten individuals performing ten actions, with similar poses across actions analyzed statistically by comparing the first action’s results with the others, as illustrated in Figure 4. Table 1 details the registration outcomes for pose pairs 1–2, 1–3, …, 1–10, using “Cor.” and “Correct Cor.” as shorthand for Correspondence and Correct Correspondence. As shown in Table 1, the pose pairs 7 and 8 output less correct correspondence compared with the other pose pairs. Pose pair 7 is the motion between pose 1 and 8 in Figure 4, which involves tremendous motion of both the upper and lower limbs, while the pair with the fewest correspondences is 1–9, which corresponds to pose pair 8 in Table 1. This is due to the symmetric nature of the human body. When the body undergoes a 180-degree orientation change, the correspondences cannot be correctly evaluated using geodesic-distance-based spatial compatibility.

Sparse matching experiments used the first mesh of each individual as the source and the remaining nine poses as targets, employing the DP algorithm and the GBMAC algorithm for generating matches. Initial pairs were formed using SHOT features and feature similarity.

The DP algorithm reduced initial matches based on a consistency threshold $c_{0}$ and a region radius σ set at 0.5 and 0.05 m, respectively. The GBMAC algorithm identified matches by finding similar points between source and target data, then constructing a spatial compatibility graph and locating matching cliques.

**Figure 5:**
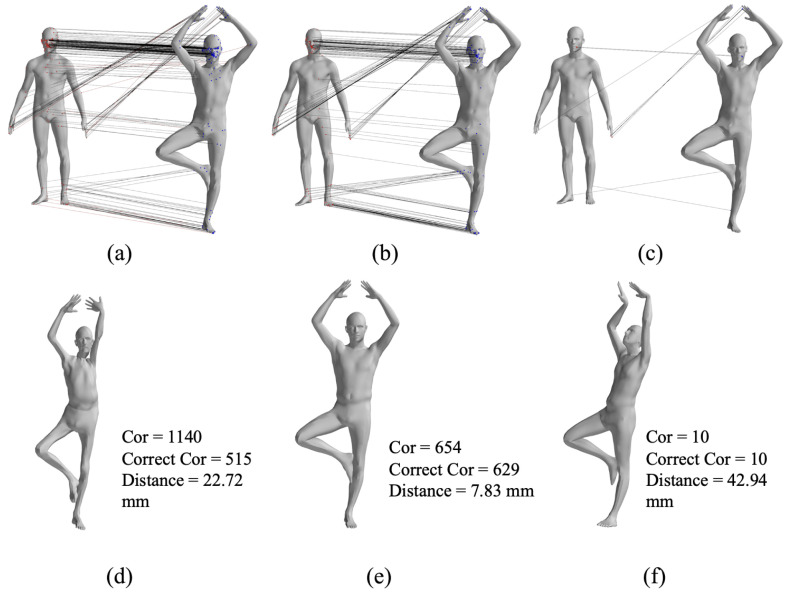
SMPL model from different maximal cliques, (**a**–**c**) are the three maximal clique sets after searching and (**d**–**f**) are the final results after non-rigid registration results with the correspondences from each set.

The GBMAC generated hypotheses through maximal clique searching and validated them with nonlinear optimization, resulting in the best matching sets. Figure 5 illustrates the maximal clique matching point sets and corresponding SMPL models for the first individual’s 1–7 pose pair, with the GBMAC producing 654 pairs, 629 of which were correct, and an average distance of 7.83 mm between the SMPL model and the data.

**Figure 6:**
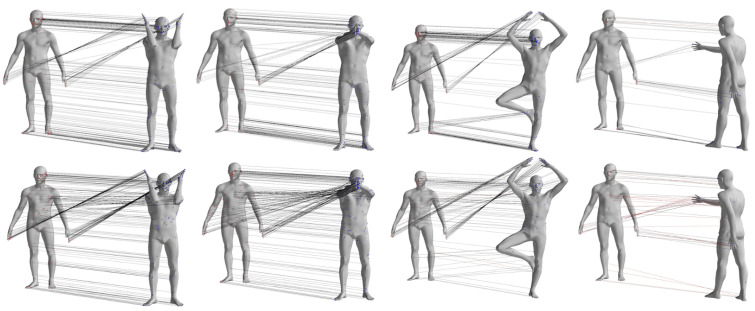
Qualitative comparison of matching pairs of GBMAC and DP algorithms obtained from the FAUST dataset.

To provide a visual comparison of the matching results obtained by the two different methods, Figure 6 shows the sparse matching results for pose pairs 1–4, 1–7, 1–8, and 1–9 of individual 00032 from the FAUST dataset. Other data pairs, where the pose differences between source and target data are small for the selected pose pairs, are not displayed. In the figure, the left column represents the matching pairs obtained by the GBMAC algorithm presented in this paper, while the right column shows the results obtained by the DP algorithm. The lines between points illustrate the matching relationships provided by the algorithms, with red lines indicating results where the distance between matching pairs exceeded 5 cm when compared to actual matching point distances.

From Figure 6, it can be observed that the GBMAC can achieve sparse matching results similar to the DP algorithm when there is minimal motion in the body’s torso. However, when the body undergoes more substantial global movement (as seen in pose pair 1–9, fourth row of the figure), the GBMAC yields higher-quality point-to-point matches.

#### 4.2.2. CAPE Dataset Evaluation

**Figure 7:**
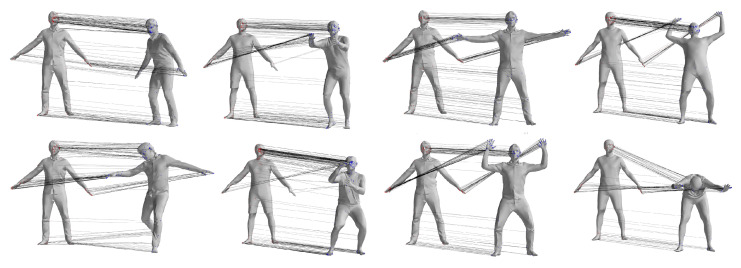
Qualitative comparison of sparse correspondence from the CAPE dataset.

The CAPE dataset consists of 15 individuals wearing different types of clothing while performing various actions. For this experiment, individual 00096 was selected, and 20 poses from 10 action sequences were chosen for non-rigid registration and human pose estimation which included Soccer, Chicken Wings, Basketball, Squats, Flying Eagle, Punching, Bend Back and Front, ATU, and Pose Model. The same algorithms used for the FAUST dataset were employed to process these data. Table 2 details the registration results for the pose pairs in actions 1–10. Figure 7 provides visual representations of the matching results between the two pose pairs from four different action categories.

#### 4.2.3. Comparison Between First-Order and Second-Order Methods

**Figure 8:**
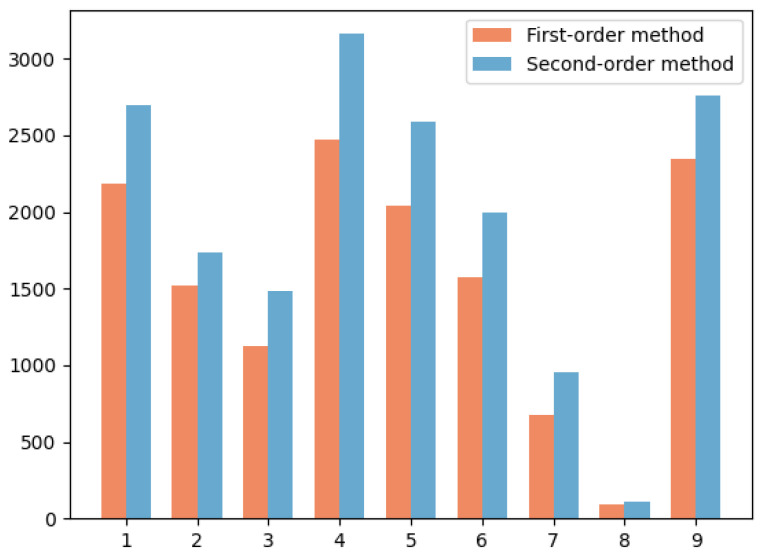
Comparison of the first-order and second-order methods applied to the FAUST dataset.

In order to illustrate the advantages and effectiveness of second-order methods over first-order methods, we compare the number of correct matches obtained by the two methods from the FAUST dataset in Figure 8. The result shows that the second-order method can produce more correct correspondence for the nine pose pairs. As a matter of fact, the first-order spatial compatibility can be disturbed by the ambiguity problem, which refers to situations where the clustering algorithm has difficulty determining the correct cluster assignments due to overlapping or unclear cluster boundaries. The second-order spatial compatibility measure can decrease the likelihood of the outlier being included in the consensus set and thus minimize the ambiguity issue. Consequently, by tackling this challenge, the second-order method has the potential to deliver superior performance in clustering endeavors.

#### 4.2.4. Time Consumption Analysis

To account for the real-time capabilities of our method, we evaluated the time consumption for each step of the experiments performed on the FAUST dataset on a desktop PC with an Intel i9-9900K CPU (Intel Corporation, Santa Clara, CA, USA). Table 3 details the averaged runtime of the four processes in our method performed on the FAUST dataset. During the experiments, the maximal clique search is the most time-consuming process, taking around one minute using the igraph library. The runtime of the maximal clique search is related to the number of matches, which can be controlled by the compatibility threshold. In the experiments, the threshold was set to 0.99, and, for slight motions which have a large number of correspondences, the value was set to 0.999 to control the number of input matches.

### 4.3. Non-Rigid Registration Evaluation

In this section, the effectiveness of the method presented in this paper is quantitatively and qualitatively evaluated for pose estimation. The results are compared with those of the GBCPD [36], N-ICP [37], RPTS [12], Fast RNRR [13], and DP-SMPL [22,35] models for the FAUST and CAPE datasets.

For quantitative evaluation, the Root Mean Square Error (RMSE) between the results of different algorithms and the ground truth is computed using the following formula:

(13) $$\mathrm{RMSE}=\sqrt{\frac{\sum_{v_{i}\in V}e_{i}^{2}}{\left|V\right|}}$$

Here, $e_{i}=∥v_{i}-v_{i}^{gt}∥$ represents the distance error between the registered points and their corresponding ground truth points, and V denotes the set of points being evaluated.

#### 4.3.1. FAUST Dataset Evaluation

**Figure 9:**
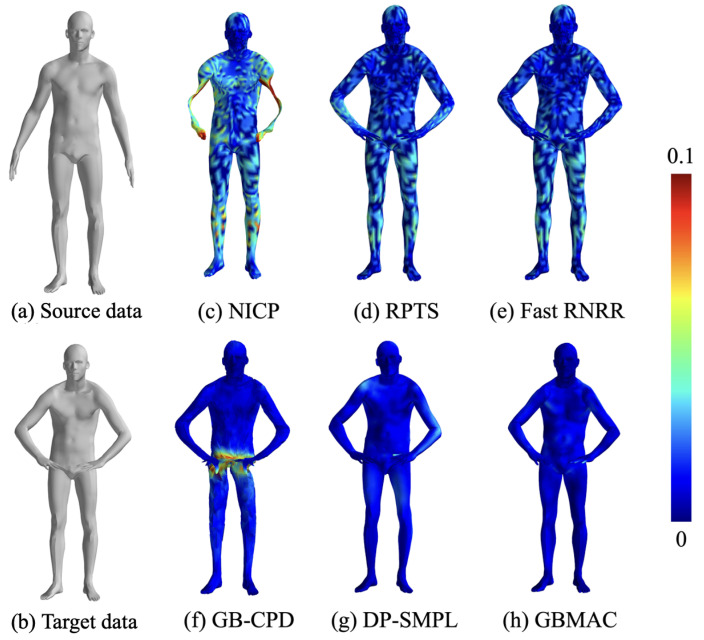
Registration results of different algorithms for the FAUST dataset.

This section compares the non-rigid human body pose registration results obtained by various algorithms from the FAUST dataset. Figure 9 displays the 0–1 pose pair, with source and target data in (a) and (b) and registration outcomes in (c)–(h), using a color gradient to indicate errors (0 to 0.1 m). From the figure, we can see that the NICP algorithm tends to cause local distortions with large movements. Non-model-based methods like RPTS and Fast RNRR perform well with accurate matching points. The experiment used DP-generated matching pairs, which were less accurate than manually selected points. The GBCPD algorithm combines geodesic and Euclidean distances, leading to abdominal misalignment with hand movements. DP-SMPL, using DP-generated points and model-based optimization, performs well with accurate matches but suffers from mismatches.

Table 4 quantitatively analyzes pose matching across algorithms. Model-free methods like Fast RNRR and DP-SMPL perform well on simple pose variations but struggle with more complex ones, where model-based methods excel due to strong priors. The GBMAC, introduced here, leverages model-based priors and auto-filters matching pairs for good results across pose pairs, with an overall RMSE of 8.86 mm for the FAUST dataset.

#### 4.3.2. CAPE Dataset Evaluation

**Figure 10:**
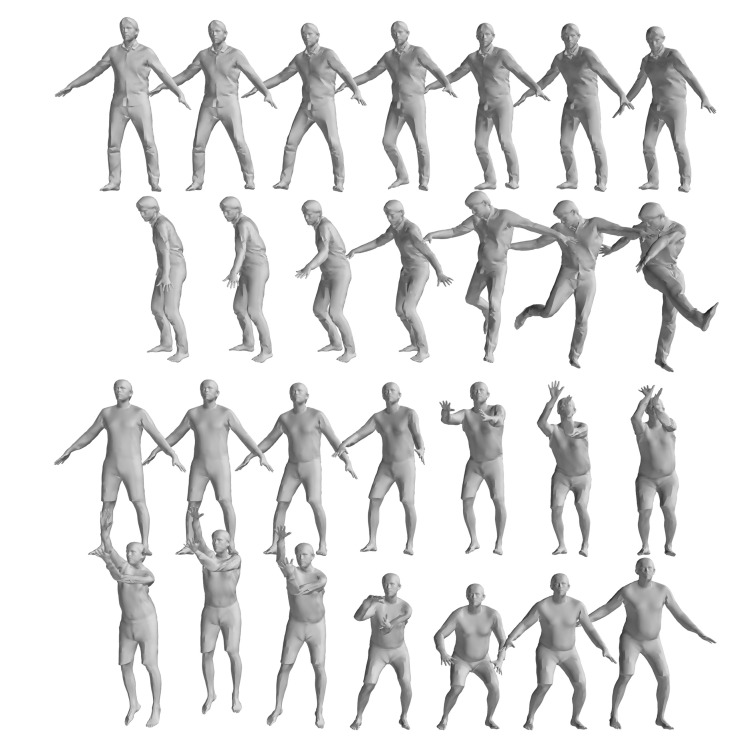
The non-rigid registration results for Soccer, Basketball, and Flying Eagle pose series from the CAPE dataset.

This section presents an experimental comparison and analysis of non-rigid human body pose registration results using different algorithms on the CAPE dataset. Compared to the FAUST dataset, the CAPE dataset offers richer data on human body pose variations, and the subjects in the CAPE dataset are dressed, making the data more similar to real-world scenarios. The experiments compared the non-rigid pose registration results for the individual numbered 00096 in the CAPE dataset across 10 different actions. The GBMAC results for the “Soccer”, “Basketball”, and “Flying Eagle” action sequences are shown in Figure 10.

From the figures, it is evident that the CAPE dataset includes more complex human body deformation actions, making the registration process more challenging. Table 5 lists the errors in matching results for different algorithms in various action groups. It can be observed that, for complex human body deformations, both BCPD and NICP algorithms fail to yield satisfactory registration results. RPTS, Fast RNRR, and DP-SMPL, on the other hand, achieve better matching results in simpler action sequences (groups 2 and 5). The GBMAC algorithm introduced in this paper outperforms other algorithms in most action groups, with an average matching error of 22.70 mm across all action groups.

## 5. Conclusions

In this paper, we introduced the GBMAC algorithm for non-rigid human point cloud registration, designed to handle deformations by building a second-order spatial compatibility graph using geodesic distances. The algorithm performs a maximal clique search to identify sparse correspondences and generates multiple sets of matching pairs. In the optimization stage, the maximal cliques are evaluated based on the root mean square distance error of neighboring points, ultimately producing an effective set of matching pairs and human pose alignments.

Our method was rigorously tested on the FAUST and CAPE datasets through both sparse matching and non-rigid registration experiments. In generating matching pairs, the GBMAC outperformed other methods, providing superior constraint relationships for subsequent tasks. Specifically, our approach achieved non-rigid matching accuracies of 8.86 mm on the FAUST dataset and 22.70 mm on the CAPE dataset, surpassing existing algorithms.

The practical benefits of the proposed GBMAC algorithm are particularly evident in real-world applications involving non-rigid point cloud registration across consecutive frames. In scenarios where device frame rates are limited and rapid human motion causes significant movement between consecutive frames, the accuracy of other algorithms often deteriorates significantly. However, the GBMAC algorithm excels in these challenging conditions, maintaining high accuracy even during large-scale movements, making it highly suitable for dynamic environments and real-time applications.

However, the efficiency of the proposed algorithm needs to be enhanced to meet real-time performance requirements in practical applications. The most time-consuming part is the maximal clique search process, which remains a key research focus for further improvement. Additionally, the non-rigid registration results are highly affected by the directional change of human orientation. This direction information could be further explored to align the human orientation before non-rigid registration.

Despite these challenges, the GBMAC algorithm demonstrates strong potential for advancing non-rigid registration tasks. Future work will focus on optimizing the algorithm’s efficiency, particularly in the maximal clique search process, and improving its scalability to handle more dynamic and complex deformations across various application scenarios.

## Figures and Tables

**Table 1:** Results comparison of sparse correspondence in FAUST dataset.

Pose Pair	Initialization		Diffusion Pruning		GBMAC	
	Cor.	Correct Cor.	Cor.	Correct Cor.	Cor.	Correct Cor.
1	2985	424	420	408	2773	2700
2	3437	945	943	941	1762	1738
3	3182	804	782	776	1519	1483
4	3492	989	984	981	3224	3163
5	3421	1028	1028	1025	2635	2590
6	2977	589	580	567	2031	1998
7	2572	93	91	68	982	953
8	2297	20	36	6	146	109
9	2976	491	476	461	2812	2758
Ave.	3145	704	699	691	1987	1944

**Table 2:** Results comparison of sparse correspondence in CAPE dataset.

Pose Pair	Initialization		Diffusion Pruning		GBMAC	
	Cor.	Correct Cor.	Cor.	Correct Cor.	Cor.	Correct Cor.
1	2670	295	311	281	895	836
2	3105	544	550	531	1288	1169
3	3013	612	621	598	1619	1509
4	3251	774	772	758	1269	1175
5	3054	356	363	340	1073	953
6	2725	320	332	303	1282	1170
7	2645	237	252	222	1020	908
8	2939	703	707	690	1189	1131
9	2553	167	182	149	901	820
10	2395	87	114	78	298	248
Ave.	2835	410	421	395	1084	992

**Table 3:** Averaged runtime for 100 launches on FAUST datasets.

Process	Time (s)
Initial Correspondences	12.6
Graph Construction	18.8
Maximal Clique Search	67.5
Hypothesis Evaluation	21.7
Total	120.6

**Table 4:** Quantitative comparisons of non-rigid registration for the FAUST dataset (mm), the bold are the best results among these methods.

Pose Pairs	GBCPD	NICP	RPTS	Fast RNRR	DP-SMPL	GBMAC
1	56.59	44.59	11.97	11.04	14.46	**8.03**
2	23.20	19.73	10.11	9.65	**5.93**	7.29
3	286.19	65.13	11.34	12.40	9.58	**8.59**
4	32.33	27.23	10.08	9.98	**6.42**	7.06
5	17.60	15.51	9.35	9.52	**6.33**	6.94
6	406.32	50.27	10.87	10.33	24.53	**8.13**
7	434.26	157.49	35.07	31.86	40.54	**9.72**
8	241.91	220.24	183.95	228.74	70.03	**15.79**
9	438.50	59.34	10.89	11.94	18.94	**8.19**
Ave.	215.21	73.28	32.63	37.27	21.86	**8.86**

**Table 5:** Quantitative comparisons of non-rigid registration for the CAPE dataset (mm), the bold are the best results among these methods.

Pose Series	BCPD	NICP	RPTS	Fast RNRR	DP-SMPL	GBMAC
1	65.71	225.82	113.92	100.07	137.99	**20.71**
2	132.79	71.70	22.84	22.41	**20.91**	23.71
3	157.79	190.16	131.03	113.31	120.20	**15.15**
4	180.07	71.13	51.45	46.15	**11.33**	12.97
5	108.45	138.98	19.43	**17.01**	39.95	17.84
6	148.32	165.45	58.89	59.30	70.45	**19.22**
7	289.61	245.75	231.03	240.29	225.74	**24.60**
8	150.21	96.44	34.21	32.80	**15.88**	20.19
9	110.17	284.97	198.32	204.83	249.95	**21.82**
10	446.41	457.97	409.23	411.68	322.52	**50.82**
Ave.	178.95	194.84	127.04	124.78	121.49	**22.70**

## Data Availability

Data are contained within the article.

## Abbreviations

The following abbreviations are used in this manuscript:

SMPL	Skinned Multi-Person Linear model
ICP	Iterative Closest Point
CPD	Coherent Point Drift
ARAP	As Rigid As Possible
FHPF	Fast Point Feature Histogram
SHOT	Signature of Histograms of Orientations
SC	Spatial Compatibility
GBMAC	Geodesic-Based Maximal Clique
